# Infectious Diseases Society for Obstetrics and Gynecology

**DOI:** 10.1155/S1064744996000014

**Published:** 1996

**Authors:** Sebastian Faro

**Affiliations:** Department of Gynecology and Obstetrics The University of Kansas School of Medicine Kansas City KS USA


					Infectious Diseases in Obstetrics and Gynecology 4:1 (1996)
(C) 1996 Wiley-Liss, Inc.
Infectious Diseases Society for Obstetrics
and Gynecology
Infectious Diseases in Obste#ics and Gynecology is proud to be the first journal to publish
the abstracts of the Infectious Diseases Society for Obstetrics and Gynecology
(IDSOG). In this issue are the abstracts of their forthcoming 1996 Annual Meeting
and Symposia to be held August 14-17, 1996, at the Hyatt Regency Beaver Creek
in Beaver Creek, Colorado.
Their scientific meeting and symposium include nationally and internationally
known obstetricians, gynecologists, pediatricians, and research scientists offering re-
search and clinical papers; invited speakers; and poster sessions. The broad-based
symposium, "Infections and Preterm Delivery," on Wednesday, August 14, will cover
the mechanisms of infection that cause preterm labor, relationship of lower- and
upper-genital-tract infections, similarities in the cytokine and endocrine response to
infection between the primate model and the human, and expected fetal immune
and placental cellular responses to present and future treatment strategies. The presen-
tation of abstracts reflecting a wide range of subjects will take place Thursday
through Saturday.
Publishing these abstracts is part of our effort to disseminate the information
obtained by investigators seeking answers to questions regarding infectious diseases
occurring in females. It also enables us to learn from the work of other investigators
and to collaborate on problems of shared interest.
Again, we are extremely grateful to be afforded this opportunity to interact with
this very prestigious and learned society.
Sebastian Faro, M.D., Ph.D., Editor-in-Chief
Department ofGynecology and ObstetrCcs
The University ofKansas
School ofMedicine
Kansas City, KS
Editorial
Received June 4, 1996
Accepted June 10, 1996

				

